# Radiographic and clinical outcomes of C1-C2 intra-articular screw fixation in patients with atlantoaxial subluxation

**DOI:** 10.1186/s13018-018-0985-9

**Published:** 2018-10-29

**Authors:** Hiroshi Uei, Yasuaki Tokuhashi, Masafumi Maseda

**Affiliations:** Department of Orthopaedic Surgery, Nihon University School of Medicine, 30-1 Oyaguchi-kamicho, Itabashi-ku, Tokyo, 173-8610 Japan

**Keywords:** Interference screw, Hook-rod constructs, Atlantoaxial subluxation, Vertebral artery injury

## Abstract

**Background:**

The Magerl and Goel-Harms techniques have been reported to produce excellent treatment outcomes in cases of atlantoaxial subluxation, but they also carry a risk of vertebral artery injuries. In order to completely prevent such injuries, we developed a surgical procedure, involving bone grafting between the C1 posterior arch and C2 lamina with clamp- or hook-and-rod-based fixation combined with the insertion of an interference screw into the posterior atlantoaxial joint.

**Methods:**

This was a retrospective single-center study. The subjects were 58 patients in whom atlantoaxial subluxation was treated with the abovementioned procedure after 1995 (33 patients with rheumatoid arthritis (RA group) and 25 patients without rheumatoid arthritis (non-RA group)). The clinical outcomes and imaging findings of anterior subluxation at ≥ 2 years after surgery were compared between the RA and non-RA groups.

**Results:**

No vertebral artery injuries occurred during surgery. Seven and two patients died during the follow-up period in the RA and non-RA groups, respectively, but none of these deaths were associated with surgery. At ≥ 2 years after surgery, the visual analogue scale score, Japanese Orthopaedic Association score, and Ranawat classification had significantly improved in both groups (*p* < 0.001). Radiologically, bone fusion was noted in all patients. Significant changes in the atlas-dens interval (ADI) were seen immediately after surgery in both groups (*p* < 0.001). In the non-RA group, significant changes in the corrected atlantoaxial height were observed immediately after surgery (*p* < 0.01), and loss of correction was seen at the final follow-up, but it was not significant (*p* = 0.1965). No significant changes were noted in any other parameter. Regarding the postoperative alignment of the cervical spine, lordosis tended to decrease, but additional surgery was only performed in one patient, who had developmental stenosis at the mid-lower level and belonged to the RA group. No reoperations due to fused adjacent segmental disease or exacerbated curvature were required.

**Conclusion:**

In the present study, no vertebral artery injuries occurred during surgery, and no major perioperative complications developed. Favorable clinical outcomes were observed at ≥ 2 postoperative years although the patients’ diseases varied. This procedure produced superior outcomes, especially in terms of spinal correction and the maintenance of the ADI.

## Background

Various surgical procedures have been developed as treatments for atlantoaxial instability, including atlantoaxial subluxation [1–5], and they all have advantages and disadvantages [6–8]. The Magerl [5, 9] and Goel-Harms techniques [10–12] have been reported to produce favorable fixation and superior treatment outcomes, and so these have become the standard surgical procedures for atlantoaxial subluxation [13–15]. On the other hand, these approaches require supportive techniques, such as fluoroscopy, and technical skill, and the risk of vertebral artery injuries is always present [16].

Thus, we have developed a method for inserting interference screws into the atlantoaxial joint without penetrating the atlantoaxial joint [17, 18]. The radiographic and clinical outcomes of this surgical procedure were investigated in patients with and without rheumatoid arthritis.

## Methods

### Patients

The subjects were 58 patients with atlantoaxial subluxation who were treated with our novel method. There were 33 and 25 patients with and without rheumatoid arthritis, respectively (the RA and non-RA groups, respectively). The non-RA group was comprised of seven patients with idiopathic subluxation, seven patients with traumatic subluxation, five patients with os odontoideum, four patients with Klippel-Feil syndrome, and two patients with athetoid cerebral palsy. The sex, age, pathology, operative time, amount of intraoperative blood loss, and duration of the postoperative follow-up period at ≥ 2 years after surgery are shown in Table 1. Only the sex ratio and the frequencies of each pathological type differed significantly between the groups.

**Table 1:** Breakdown of disease

	Rheumatoid arthritis (*n* = 33)	Non-rheumatoid arthritis (*n* = 25)	*p* value
Male:female	8: 25	16: 9	0.0023*
Age (years)	63.6 ± 11.5 (27–76)	55.6 ± 10.5 (13–72)	0.1267
Pathology	Rheumatoid arthritis 33	Idiopathic 7	
		Traumatic 7	
		Os odontoideum 5	
		Klippel-Feil 4	
		Athetoid cerebral palsy 2	
Anterior atlanto-axial subluxation	30	22	0.4168
Anterior atlanto-subluxation + vertical subluxation	2	0	0.8882
Posterior atlanto-axial subluxation	1	3	0.4168
With subaxial lesion	8	3	0.4011
Operating time (min)	166.2 ± 43.5 (114–260)	189.0 ± 52.8 (100–277)	0.1750
Bleeding (mL)	242.1 ± 268.0 (15–1255)	178.9 ± 185.5 (36–609)	0.3750
Follow-up period (months)	88.33 ± 60.2 (24–210)	109.5 ± 81.7 (24–262)	0.4474

*Significant difference (*p* < 0.05)

A unilateral/bilateral high-riding vertebral artery (HRVA) was defined as a C2 isthmus height of ≤ 5 mm and/or an internal C2 height of ≤ 2 mm on a reconstructed sagittal computed tomography (CT) image obtained 3 mm lateral to the cortical margin of the spinal canal wall. In this series, HRVA were found in 12 (20.7%) patients. Of these, 3 (5.2%) patients had bilateral HRVA.

All 48 patients had occipital and neck pain at ≥ 2 years after surgery, and numbness of the upper limbs and lower limb symptoms were noted in 30 and 23 patients, respectively.

### Surgical procedure

#### C1-C2 intra-articular screws

**Fig. 1:**
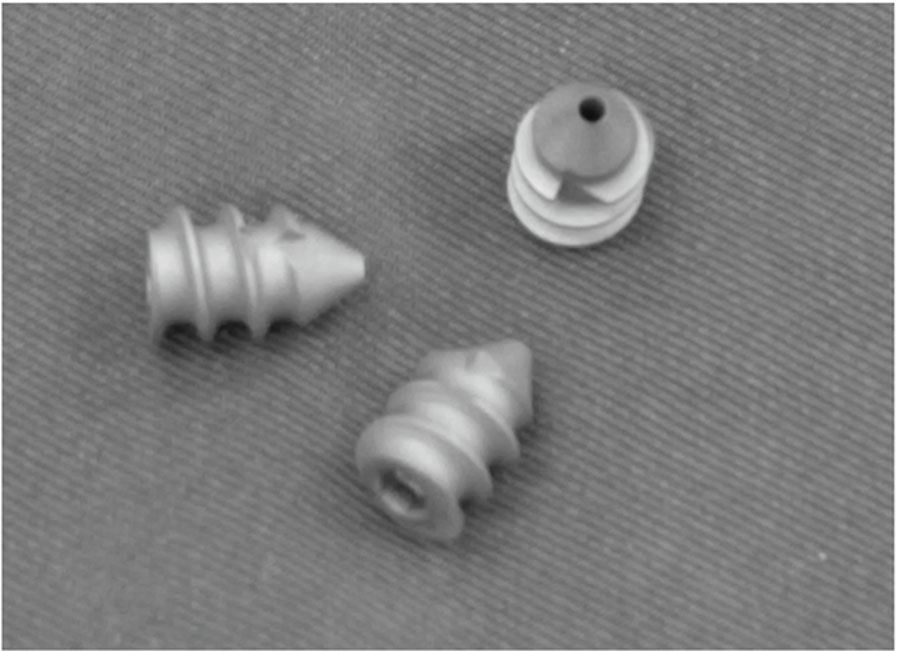
The C1-C2 intra-articular screw. The interference screw that was inserted into the atlantoaxial joint had a hollow structure, which allowed a guidewire to be inserted through it. It measured 5.6 or 6.5 mm in diameter and 8 or 10 mm in length

The interference screws were hollow, which allowed guidewires (Intra-articular spacer®, KISCO Co. Kobe, Japan) to be inserted through them. They were made of a titanium alloy and came in 4 types (diameter 5.6 or 6.5 mm; length 8 or 10 mm) (Fig. 1). The size of the screw was chosen based on CT measurements so that the screw would remain in the surface of the atlantoaxial joint while ensuring that there was a sufficient margin around it.

#### C1-C2 intra-articular screw fixation

**Fig. 2:**
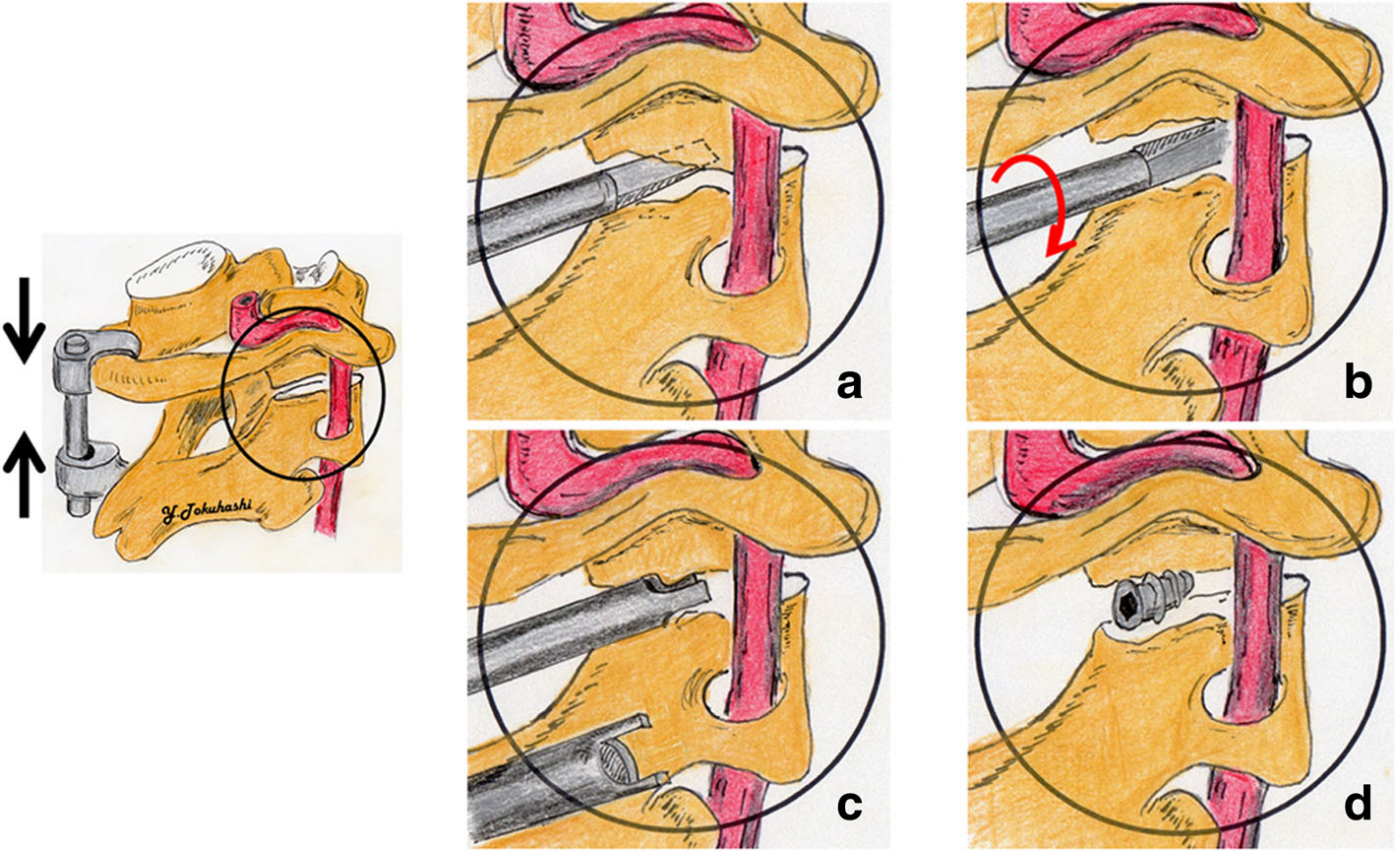
The guide used to insert screws into the atlantoaxial joint. **a** An elevator was inserted into the atlantoaxial joint. **b** The inserted elevator was rotated 90°, and the atlantoaxial joint was lifted up. **c** The nail region of the fork-shaped (two-pronged) cylindrical insertion guide was inserted through the elevator into the atlantoaxial joint. **d** An intra-articular screw was inserted into the atlantoaxial joint through the fork-shaped cylindrical insertion guide

**Fig. 3:**
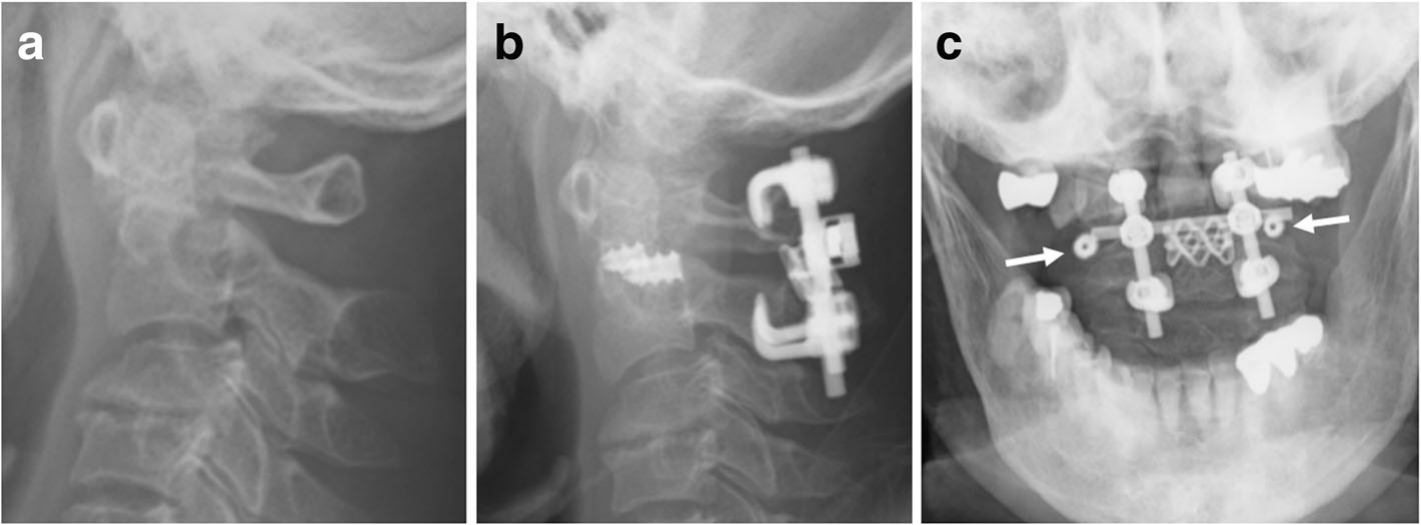
C1-C2 intra-articular screw fixation with a titanium mesh cage: an 80-year-old female with an odontoid fracture. **a** Lateral view on a plain radiograph before surgery. **b** Lateral view on a plain radiograph immediately after surgery. An intra-articular screw was inserted into the atlantoaxial joint. Hook-and-rod fixation was applied to the posterior region. **c** Anterior view on a plain radiograph immediately after surgery. The region between the posterior arch of the C1 vertebra and the lamina of the C2 vertebra was filled with a titanium cage and grafted bone. White arrow: the intra-articular screw in the atlantoaxial joint

First, the neurovascular bundle was pulled toward the cranial side to expose the posterior atlantoaxial joint. Then, a thin elevator was inserted into the posterior atlantoaxial joint, and bilateral joint release was performed as much as possible. Next, the atlantoaxial subluxation was reduced by applying pressure in the direction shown by the arrow between the vertebral arches on the opposite side using a clamp or hook-and-rod system (OASYS® hook and rod, Stryker Co.) to achieve temporary fixation (Fig. 2, left). An interference screw was inserted and buried in the exposed posterior atlantoaxial joint to achieve fixation. The screw insertion site in the posterior atlantoaxial joint was located at the center of the articular surface, and the interference screw was inserted perpendicular to the articular surface until the caudal edge of the screw was located 1 mm from the articular surface. Posterior stabilization was applied between the posterior arch of the C1 vertebra and the lamina of the C2 vertebra on the screw fixation side, using semi-layer iliac grafting and a hook-and-rod system. Bone grafting with a titanium mesh cage packed with an autograft and hydroxyapatite was applied in the 8 most recent patients (Fig. 3) [19]. This procedure was then performed on the opposite side. In recent cases, a fork-shaped (two-pronged) insertion guide (Asami® device, Kisco Co., Kobe, Japan) has been used under fluoroscopy, which made the insertion procedure easier and increased its reliability (Fig. 2). After surgery, the patients wore a simple cervical spine collar for 2–3 months. In one case, a concomitant spinal lesion (a sub-axial lesion), which was located at the mid-lower cervical level, was treated with decompression and fusion at the same time as the aforementioned surgical procedure (Table 1).

### Study measures

Pain was evaluated using a visual analogue scale (VAS). Neurological manifestations were evaluated using the Japan Orthopaedic Association score (JOA score) for cervical myelopathy (a 17-point system) [20] and the Ranawat classification of neurological deficits [3] and were classified into the following four classes: class I: no neurological deficits; class II: subjective weakness, hyperreflexia, dysesthesia; class IIIa: objective weakness, long tract signs, able to walk; and class IIIb: objective weakness, long tract signs, unable to walk.

### Radiological assessment

Radiographically, bone union was considered to have occurred when no mobility was observed between the C1 and C2 vertebrae on dynamic plain radiographs and when continuity of the trabeculae of the grafted bones was observed between the posterior arch of the C1 vertebra and the lamina of the C2 vertebra on lateral-view plain radiographs and/or reconstructed CT. We observed bone union at approximately 3 months after surgery.

**Fig. 4:**
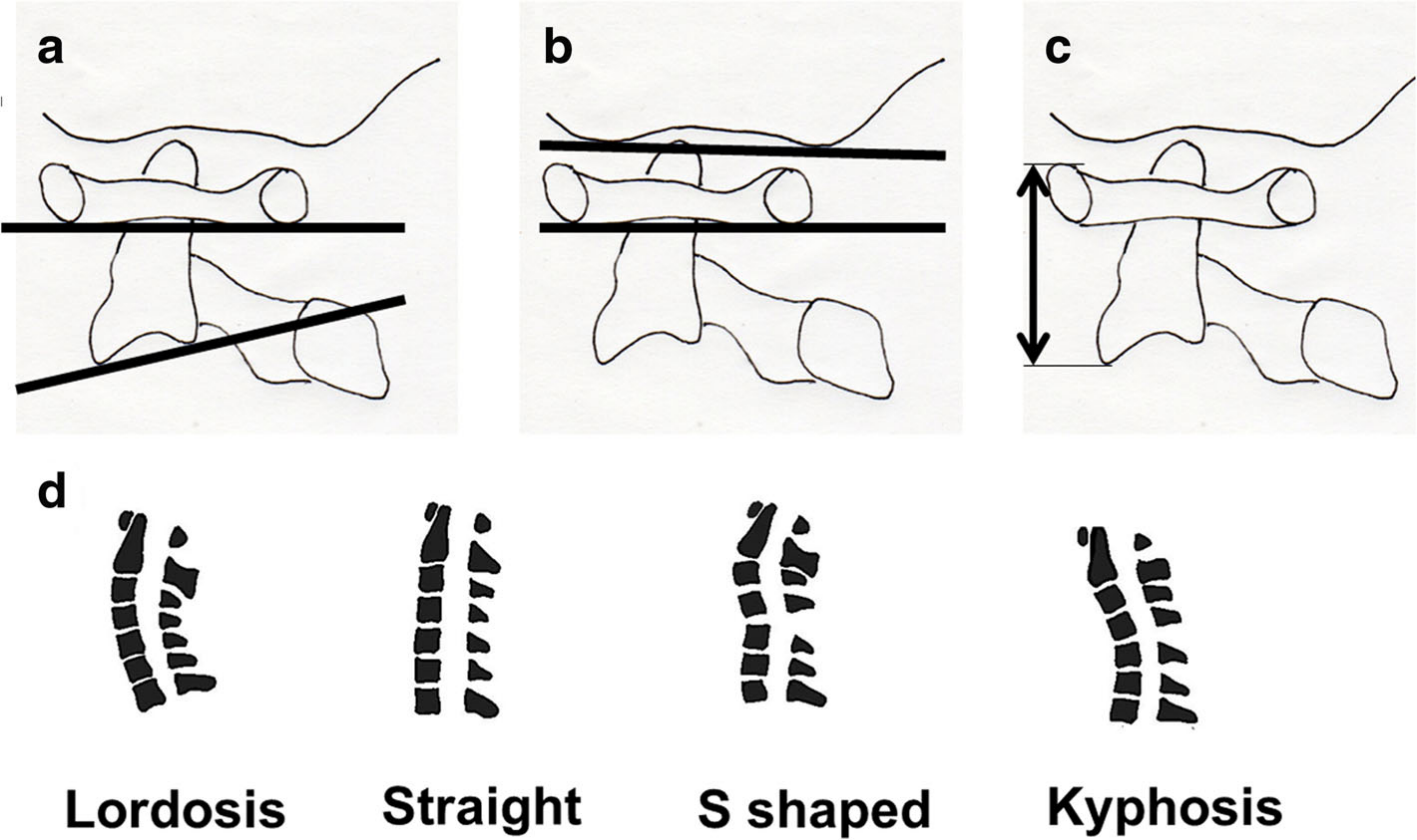
Radiographic parameters. **a** Atlantoaxial angle (A-A angle, the C1-C2 angle). The atlantoaxial fixation angle, which is the angle formed by the line connecting the lower margins of the anterior and posterior arches of the C1 vertebra and the lower margin of the C2 vertebra. **b** Range of motion between the occipital bone and the atlas (ROM of O-C1). On the lateral view of the cervical spine, the range of motion of the angle formed by the line connecting the anterior and posterior margins of the foramen magnum and the line connecting the anterior and posterior lower margins of the C1 arch, i.e., the range of motion between the occipital bone and atlas, was assessed. **c** Atlantoaxial height (A-A height). The height of the atlantoaxial joint, corrected for the distance between the upper margin of the anterior arch of the C1 vertebra and the lower margin of the C2 vertebral body, on a lateral-view plain radiograph is shown. **d** Alignment of the entire cervical spine. Lordosis, straight, s-shaped, or kyphosis

As for the examined parameters, the atlas-dens interval (ADI), the atlantoaxial angle (A-A angle: the C1-C2 angle); i.e., the angle between the line connecting the lower margins of the anterior and posterior arches of the C1 vertebra and the lower margin of the vertebral body of the C2 vertebra on a lateral-view plain radiograph (Fig. 4a) [18]; the range of motion between the occipital bone and atlas (O-C1 ROM); i.e., the change in the angle between the line connecting the anterior and posterior margins of the greater foramen and the line between the lower margins of the anterior and posterior arches of the C1 vertebra on a lateral-view plain radiograph (Fig. 4b) [18]; and atlantoaxial height (A-A height); i.e., the distance between the upper margin of the anterior arch of the C1 vertebra and the lower margin of the C2 vertebra (Fig. 4c) [18] were evaluated. Three-dimensional CT-based mensuration was considered to be a more accurate way of assessing A-A height than plain radiograph-based mensuration, but the former technique could not be performed during the 2 years after surgery in all cases. Therefore, we measured it on lateral-view plain radiographs in all cases. The alignment of the entire cervical spine was also evaluated and classified into the following four types: lordosis, straight, s-shaped, and kyphosis (Fig. 4d).

### Statistical analysis

For comparisons between two items/groups, the *t* test, Welch’s method, the paired *t* test, or the Mann-Whitney *U* test was used. For comparisons among multiple items/groups, analysis of variance (ANOVA), the Tukey test, or the Kruskal-Wallis test was used. All statistical analyses were performed using StatMate V® (Atoms Co.; Tokyo, Japan), and *p* values of < 0.05 were regarded as significant.

## Results

### Surgical results

The mean operative time was 166.2 ± 43.5 min (range 114–260 min) in the RA group and 189.0 ± 52.8 min (100–277 min) in the non-RA group (*p* = 0.1750). The mean amount of intraoperative blood loss was 242.1 ± 268.0 mL (range 15–1255 mL) and 178.9 ± 185.5 mL (range 36–609 mL) in the RA and non-RA groups, respectively (*p* = 0.3750). No vertebral artery injuries occurred during surgery in any case. A reoperation was required in one patient due to implant dislodgement, but no perioperative complications that required reoperations, such as infections, occurred. Asymptomatic screw displacement was noted in three patients, but no such incidents occurred after the introduction of the insertion guide and fluoroscopy. Regarding delayed complications, no infections or instrumentation failure occurred, and a reoperation was only required in one patient, in whom laminoplasty was performed for developmental stenosis of the mid-lower cervical spine. Transient occipital numbness, which was assumed to have been caused by C2 root damage, occurred in seven patients. However, no patients suffered permanent occipital numbness.

### Clinical results

Seven patients (21.2%) died during the follow-up period in the RA group (2 and 3 patients died of pneumonia within 2 years and after 2 years, respectively, and 1 patient each died of sepsis and colon cancer). No patients died within the first postoperative year. All seven of the aforementioned patients returned to active daily life after the operation, and none of them became bedbound. In the non-RA group, 2 patients (8.0%) died within the first 2 postoperative years (1 each died of aortic stenosis and pneumonia; no patients died after 2 postoperative years). They also returned to active daily life after the operation, and neither of them became bedbound.

Improvements in symptoms were seen in all patients, including those who died, and the VAS score, JOA score, and Ranawat classification of neurological deficits had all significantly improved at ≥ 2 years after surgery in both groups (*p* < 0.001). No significant differences in outcomes were noted between the two groups (Table 2).

**Table 2:** Clinical results

	Rheumatoid arthritis group (*n* = 32)	*p* value between before and after	Non-rheumatoid arthritis group (*n* = 22)	*p* value between before and after	*p* value between RA and non-RA
VAS					
Before	62.56 ± 18.28 (30–90)	< 0.001*	56.60 ± 16.76 (30–76)	< 0.001*	0.3135
Final	5.76 ± 10.44 (0–45)		5.00 ± 9.13 (0–36)		0.8258
JOA score					
Before	11.37 ± 3.78 (4–17)	< 0.001*	13.19 ± 3.45 (6–17)	< 0.001*	0.1521
Final	16.64 ± 0.79 (14–17)		16.23 ± 1.30 (13–17)		0.3224
Ranawat class	I, II, IIIa, IIIb		I, II, IIIa, IIIb		
Before	4, 13, 12, 0	< 0.001*	6, 6, 8, 3	< 0.001*	0.8840
Final	22, 7, 0, 0		19, 4, 0, 0		0.8535

*RA* rheumatoid arthritis, *VAS* visual analogue scale, *JOA* Japan Orthopaedic Association

*Significant difference (*p* < 0.05)

### Radiological results

Fusion of the posterior bone grafts was confirmed based on plain radiographs in all patients. Forty-eight patients underwent CT scans at the same time as the plain radiography, and fusion of the atlantoaxial joint was also confirmed on CT in 19 of them at ≥ 2 years after surgery [21].

In the RA group, the mean preoperative ADI was 7.93 ± 2.32 mm (3.7–13.0 mm), and it decreased to a mean of 2.47 ± 1.27 mm (range 0–5.7 mm) immediately after surgery (*p* < 0.001). The mean value at the final follow-up was 2.41 ± 1.42 mm (range 0–6.1 mm), and no changes of ≥ 2 mm in the ADI were noted during the follow-up period in any patient (*p* = 0.1480). In the non-RA group, the ADI decreased significantly from 7.90 ± 2.39 mm (5.1–14.2 mm) to 3.79 ± 1.59 mm (range 0.5–6.3 mm) immediately after surgery (*p* < 0.001), and it was 4.49 ± 2.16 mm (range 1.5–8.9 mm) at the final follow-up (*p* = 0.1965). In a comparison between the two groups, the degree of the reduction in the ADI was significantly greater in the RA group (*p* = 0.0059), and the final ADI was also significantly more favorable in the RA group (*p* < 0.001) (Table 3).

**Table 3:** Radiological results

	Rheumatoid arthritis group (*n* = 32)	*p* value between before and after	No rheumatoid arthritis group (*n* = 22)	*p* value between before and after	*p* value between RA and non-RA
Atlanto-dental interval (mm)					
Pre-operation	7.93 ± 2.32 (3.7–13)		7.90 ± 2.39 (5.1–14.2)		0.9717
Just after operation	2.47 ± 1.27 (0–5.7)	< 0.001*	3.79 ± 1.59 (0.5–6.3)	< 0.001*	0.0059*
At final follow-up	2.41 ± 1.42 (0–6.1)	0.1480	4.49 ± 2.16 (1.5–8.9)	0.1965	< 0.001*
Atlanto-axial angle (degree)					
Pre-operation	21.5 ± 9.57 (4.0–42.0)		19.14 ± 2.39 (8.0–35.2)		0.4540
Just after operation	22.3 ± 9.50 (0–36.0)	0.1796	25.07 ± 5.47 (15.2–36.1)	0.0849	0.3042
At final follow-up	20.9 ± 8.21 (3.0–36.0)	0.2437	23.0 ± 6.89 (13.4–39.2)	0.3373	0.4073
Range of motion of occipito-atlas (degree)					
Pre-operation	4.45 ± 3.59 (0–12.0)		8.25 ± 4.90 (3.0–12.0)		0.1171
At final operation	5.96 ± 5.03 (0–16.4)	0.8116	7.08 ± 6.13 (1.4–22.1)	0.6721	0.5600
Atlanto-axial height(mm)					
Pre-operation	40.87 ± 18.49 (0–16.6)		43.70 ± 4.42 (37.4–51.2)		0.0899
Just after operation	43.47 ± 6.30 (24.2–53.5)	0.1882	46.87 ± 4.49 (39.1–53.8)	0.0018*	0.0758
At final follow-up	41.24 ± 6.17 (19.8–52.2)	0.3385	44.33 ± 5.22 (37.4–53.6)	0.0019*	0.1051
Cervical alignment (C1-C7)	L, St, S, K		L, St, S, K		
Pre-operation	18, 4, 6, 0		9, 2, 9, 0		0.5690
At final follow-up	15, 4, 7, 2	0.3209	8, 2, 10, 0	0.7531	0.2563

*Significant difference (*p* < 0.05)

*L* lordosis, *St* straight, *S* S shaped, *K* kyphosis

At the final follow-up, the A-A angle ranged from 3.0–36.0° (mean 20.9 ± 8.21°) in the RA group and from 13.4 to 39.2 (mean 23.0 ± 6.89°) in the non-RA group (Fig. 4a). An A-A angle of 30° means that the line connecting the lower margins of the anterior and posterior arches of the C1 vertebra and the lower margin of the vertebral body of the C2 vertebra runs almost parallel to the axis of the atlantoaxial arches (when this angle is 30° the vertebral arches of the C1 and C2 vertebrae are nearly parallel). Angles of ≥ 31°, which are suggestive of overcorrection, were only noted in 2 (7.1%) and 2 (10%) patients in the RA and non-RA groups, respectively (Table 3).

The range of motion between the occipital bone and atlas (Fig. 4b) increased after surgery in both groups, but the increase was not significant in either group (Table 3).

**Fig. 5:**
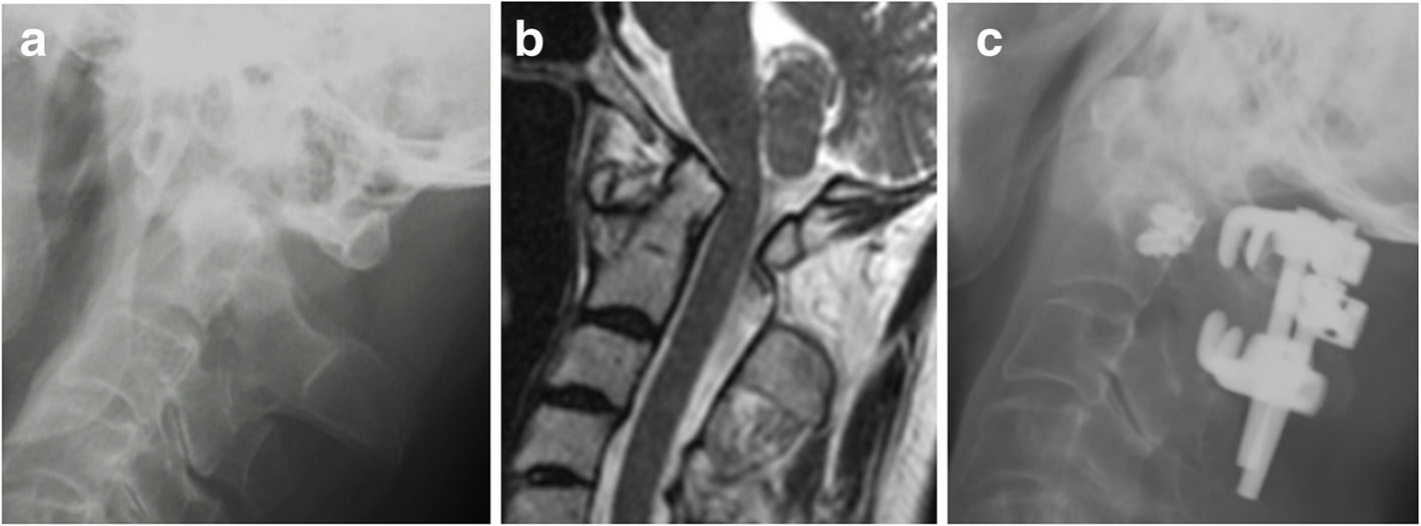
A 63-year-old female with rheumatoid arthritis and vertical subluxation. **a** Lateral view on a preoperative plain radiograph. The A1-A2 height was 30.9 mm. **b** Preoperative MRI. Vertical subluxation was noted. **c** Lateral view on a postoperative plain radiograph. The A1-A2 height had increased to 36.9 mm

The atlantoaxial height (A-A height) (Fig. 4c) increased because the height of the atlantoaxial joints increased after the insertion of the interference screw, but the increase was not significant in the RA group, and the loss of correction seen at the final follow-up was also not significant. As for symptomatic improvement and symptoms associated with the increase in the A-A height, no characteristic findings were seen in either group. In contrast, in the non-RA group the A-A height increased significantly immediately after surgery, and significant loss of correction was noted at the final follow-up (Table 3). In two rheumatoid arthritis patients with vertical subluxation, the A-A height increase seen after surgery was maintained throughout the follow-up period (Fig. 5).

Regarding the postoperative changes in the alignment of the cervical spine (Fig. 4d), lordosis slightly decreased in both the RA and non-RA groups, and kyphosis only developed in two patients (7.1%) in the RA group.

## Discussion

The surgical procedure employed in the present cases resulted in biomechanically favorable stabilization through 3-point fixation on both sides of the atlantoaxial joint and posterior arch [17], but the resultant braking force against rotation was significantly greater than that acquired by Magerl’s technique [17]. The treatment outcomes of the RA and non-RA groups did not differ significantly.

Furthermore, concerning irreducible atlantoaxial subluxation, it has been said that the Magerl method is not suitable for treating such cases because it does not involve a reduction procedure [15]. In contrast, the advantages of this operation include the fact that regardless of whether the C1-C2 section is irreducible, a reduction procedure can be performed using a hook-rod system after the release of the posterior atlantoaxial joints via their expansion. Furthermore, the use of the insertion guide made the dilation of the posterior atlantoaxial joints easy (Fig. 2b), which in turn facilitated reduction.

Another characteristic of the procedure examined in this study that has not been noted in other surgical procedures is that the height of the atlantoaxial joint can be preserved by distracting the atlantoaxial joint. This approach can be used to prevent progression from atlantoaxial subluxation to vertical subluxation or to treat mild vertical subluxation in patients with rheumatoid arthritis. Recent advances in the treatment of rheumatoid arthritis have inhibited the progression and aggravation of rheumatoid arthritis of the cervical spine, markedly reducing the number of patients who require surgery. However, in two patients with rheumatoid arthritis-induced vertical subluxation in the present study lifting up the atlantoaxial joint improved or prevented the progression of vertical subluxation (Fig. 5). Goel A (2008) also reported that inserting a spike-like implant into the atlantoaxial intervertebral joint from the posterior side was effective at lifting up the atlantoaxial joint [22, 23]. Shortening the range of fixation as much as possible is useful because it minimizes the long-term influence of the procedure on the mid-lower cervical spine. The significance of this approach will increase as measures for preventing the progression of rheumatoid arthritis advance.

Magerl’s technique has frequently been reported to carry a risk of vertebral artery injuries [13, 16, 24]. The frequency of vertebral artery injuries during Magerl’s procedure was reported to range from 4.1–8.2% [25–27], and the frequency of HRVA, which is the main cause of such injuries, was reported to range from 18 to 23% [28, 29]. Moreover, although screw fixation has recently been used to treat lateral atlas masses, the internal carotid artery is located anterior to the atlas, and so penetrating lateral atlas masses with screws is not safe [12, 30, 31]. Performing Magerl’s procedure in patients with HRVA is challenging; thus, spinal surgeons have developed various C2-fixation screws for preventing vertebral artery injuries, including short pedicle screws, pars/isthmus screws, translaminar screws, new trajectory/unilateral transarticular screws, and the hook-and-rod system [32–35].

In contrast, the insertion technique employed in the current study was safe and carried almost no risk of injuring the vertebral artery because the screw could be inserted into the atlantoaxial joint under almost direct vision. In addition, the introduction of fluoroscopy and the use of a fork-shaped (a two-pronged fork) insertion guide have facilitated reliable screw insertion. The only technical disadvantage of this approach is that it results in hemorrhaging from the venous plexus and retraction of the C2 root during the exposure of the atlantoaxial joint. The hemorrhaging could not be stopped by gradually applying coagulation hemostasis. Exposing the venous plexus via en bloc distraction using Love’s nerve retractor was effective, but the introduction of the insertion guide allowed this procedure to be completed within a short time. In addition, when hemorrhaging occurred, it could be easily stopped by compressing the hemorrhage with a hemostatic agent, such as Avitene® (Davol Inc., Woburn, MA) [17]. Concerning C2 root injuries, there were no cases of permanent occipital numbness.

Many studies have examined the A-A angle (the C1-C2 angle), which corresponds to the atlantoaxial fixation angle. Yoshimoto H et al. found that when the A-A angle was large, which is indicative of fixation having been performed in an overextended position, the mid-lower cervical spine became kyphotic, which highlights the importance of intraoperative decisions regarding the A-A angle [36]. In a study of patients with rheumatoid arthritis, Kato Y et al. reported that the postoperative C2-C7 angle was not correlated with the postoperative A-A angle, but the postoperative C2-C7 angle was influenced by the degree of correction in the A-A angle [37]. An A-A angle of 30° means that the line connecting the lower margins of the anterior and posterior arches of the C1 vertebra and the lower margin of the vertebral body of C2 runs almost parallel to the axis of the atlantoaxial arch, and the C1-C2 region is overextended when the A-A angle is > 30°, which might have a large influence on the alignment of the mid-lower cervical spine.

In our procedure, the A-A angle is ≤ 30° because the atlantoaxial joint is distracted, and so fixation is unlikely to be performed in an overextended position. Fortunately, only one patient required a reoperation due to a problem with their mid-lower cervical spine during the study period, and this case involved developmental stenosis. We were initially concerned about the potential for adjacent disc problems and kyphosis of the cervical spine, but no reoperations due to these problems were required in any patient, including in the non-RA group. It was not possible to examine the influence of this screw fixation method on the whole alignment of the cervical spine. A long-term study involving a large number of cases is required to examine the effect of the atlantoaxial fixation angle on the risk of overextension.

This study had several limitations. For example, it was not a randomized control study, and the severity of rheumatoid arthritis was likely to differ among the patients in the RA group due to advances in the drugs used to treat RA because the patients were followed for a prolonged period. Such advances might also have influenced the long-term outcomes and imaging findings of each case.

## Conclusion

The treatment outcomes of 58 patients with atlantoaxial subluxation that were treated with C1-C2 intra-articular screw fixation were investigated. No intraoperative vertebral artery injuries or major perioperative complications occurred. The patients’ clinical outcomes at ≥ 2 years after surgery were also favorable although their diseases varied. In addition, this procedure resulted in superior correction of the ADI and a greater increase in the diameter of the spinal canal. On the sagittal view of the atlantoaxial joint, limited maintenance of the angle, height, and alignment of the cervical spine was seen, but no reoperations were required due to adjacent disc problems or exacerbated curvature in any patient.

## Abbreviations

AA angle: Atlantoaxial angle
AA height: Atlantoaxial height
A-A height: Atlantoaxial height
ADI: Atlas-dens interval
JOA: Japan Orthopaedic Association
MRI: Magnetic resonance imaging
O-C1 ROM: Range of motion between the occipital bone and atlas
RA: Rheumatoid arthritis
VAS: Visual analogue scale
